# A Simulation-Based Optimization Model to Study the Impact of Multiple-Region Listing and Information Sharing on Kidney Transplant Outcomes

**DOI:** 10.3390/ijerph18030873

**Published:** 2021-01-20

**Authors:** Zahra Gharibi, Michael Hahsler

**Affiliations:** 1 Department of Management, Information Systems and Analytics, State University of New York at Plattsburgh, Plattsburgh, NY 12901, USA; 2 Department of Engineering Management, Information, and Systems and Department of Computer Science, Southern Methodist University, Dallas, TX 75205, USA; mhahsler@lyle.smu.edu

**Keywords:** simulation model, kidney acceptance, kidney allocation, multiple-region listing, information sharing

## Abstract

More than 8000 patients on the waiting list for kidney transplantation die or become ineligible to receive transplants due to health deterioration. At the same time, more than 4000 recovered kidneys from deceased donors are discarded each year in the United States. This paper develops a simulation-based optimization model that considers several crucial factors for a kidney transplantation to improve kidney utilization. Unlike most proposed models, the presented optimization model incorporates details of the offering process, the deterioration of patient health and kidney quality over time, the correlation between patients’ health and acceptance decisions, and the probability of kidney acceptance. We estimate model parameters using data obtained from the United Network of Organ Sharing (UNOS) and the Scientific Registry of Transplant Recipients (SRTR). Using these parameters, we illustrate the power of the simulation-based optimization model using two related applications. The former explores the effects of encouraging patients to pursue multiple-region waitlisting on post-transplant outcomes. Here, a simulation-based optimization model lets the patient select the best regions to be waitlisted in, given their demand-to-supply ratios. The second application focuses on a system-level aspect of transplantation, namely the contribution of information sharing on improving kidney discard rates and social welfare. We investigate the effects of using modern information technology to accelerate finding a matching patient to an available donor organ on waitlist mortality, kidney discard, and transplant rates. We show that modern information technology support currently developed by the United Network for Organ Sharing (UNOS) is essential and can significantly improve kidney utilization.

## 1. Introduction

Chronic kidney disease (CKD) is a progressive loss of kidney function over time. CKD is a worldwide health crisis as, at the moment, more than 2 million patients are suffering from end-stage renal disease (ESRD) or kidney failure. The number of patients diagnosed with ESRD is expected to increase at a rate between 5% and 7% each year [1]. At present there is no cure for kidney failure, and patients with ESRD need to receive frequent dialysis or a kidney transplant from a living or deceased donor to survive. For most patients, kidney transplantation is the preferred treatment that provides a longer life expectancy with a higher quality of life than dialysis. However, patients worldwide are faced with a chronic shortage of donor kidneys accessible for transplant.

At present in the US, close to 100,000 patients are on the waiting list, and on average, over 3000 new patients are enlisted each month. Each year, more than 4000 patients die while waiting for a lifesaving kidney transplant, and over 4000 become too sick and are removed from the waiting list. In 2019, out of 22,304, deceased donor kidneys were procured in the US, a total of 16,534 kidneys were transplanted. Despite the high demand and significant kidney shortage, approximately one in five kidneys recovered from deceased donors are discarded [2].

To understand the reasons behind such high discard rates, we need to look at the kidney allocation and offering process. There are considerable differences between living and deceased donor kidneys and between different countries. We focus here on kidneys from deceased donors in the US. The most important criteria for deceased donor kidney allocation are (1) donor-recipient medical compatibility, (2) logistical factors, and (3) the patient position on the waitlist (e.g., waiting time, points). More specifically, in the US, the United Network of Organ Sharing (UNOS) administers the Organ Procurement and Transplantation Network (OPTN) and is responsible for collecting data on both patients and donors. In addition to logistical information and waiting time, the waiting list data includes the patient’s identity, demographic factors (e.g., gender, race, age), and medical characteristics (e.g., ABO blood type, human leukocyte antigens (HLAs), panel-reactive antibody (PRA)). Similarly, to create a deceased donor database, UNOS obtains information on donor demographics, donor logistical, recovery and preservation, and donor medical characteristics.

UNOS uses a centralized computer network to connect all Organ Procurement Organizations (OPOs) and transplant centers. To allocate donated kidneys, UNOS uses its donor-recipient matching system. Each time a new deceased donor kidney is retrieved for transplantation, UNOS applies a match-run algorithm, a program that compares donor data with the active waitlisted patients’ data. A rank-ordered list of patients is generated using kidney allocation rules and policies. Factors considered in creating this list include waiting time, donor-recipient immune system compatibility, living donor priority eligibility, distance from the donor hospital, survival benefit (donor-recipient longevity matching), and pediatric status.

**Figure 1:**
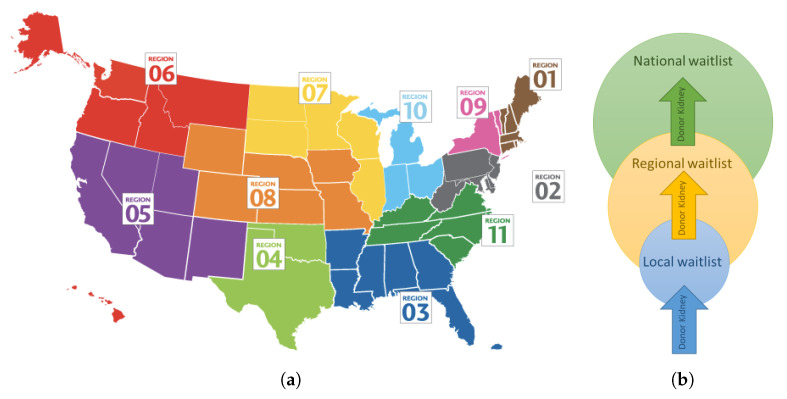
(**a**) 11 geographic regions in the US [3], (**b**) Geographical hierarchy of kidney offering process.

The complete offering process is complex and we focus here only on the main components that are necessary for the simulation model discussed in this paper. The process starts with patients listed in local OPOs (there are 58 OPOs in the US, each with its designated service area), who are medical compatible and have the highest priority on the waitlist. If the local allocation is unsuccessful, the organ is offered in the region (the US is currently divided into 11 transplantation regions) and finally nationwide. Figure 1a,b show 11 geographic regions in the US [3] and the geographical hierarchy of the kidney offering process, respectively. More details on organ procurement and allocation policy are available in [4]. One reason for prioritizing local patients in the kidney assignment process is to reduce the time between organ procurement and implantation. This time is called Cold Ischemia Time (CIT) and plays an essential role in kidney transplant outcomes [5,6].

**Figure 2:**
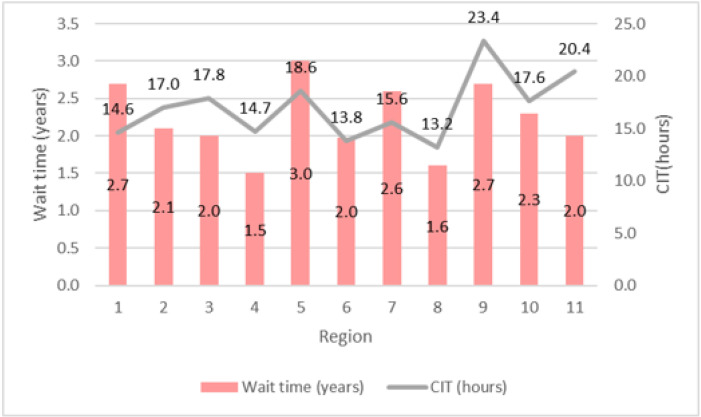
Average wait time and cold ischemia time across 11 regions in US (2019).

Figure 2 and Table 1 show regional variations in CIT, wait time, and kidney transplant outcomes across the US, respectively. There is substantial variation in deceased donor kidney wait times across the US. Multiple factors can influence a patient wait time until transplant. Besides the patient’s clinical factors such as blood type and degree of sensitization shown by PRA (panel reactive antibody), geography and patient’s place of residence has a tremendous effect on the chance of accessing a timely kidney transplant. This is important since regions with longer CIT are more likely to have lower post-transplant graft and patient survival rates. More precisely, as the results of one- and five-year patient and graft survival rates following a kidney transplant suggest, region 9 with the longest CIT among all regions has the lowest one- and five-year patient and graft survival rates among all 11 regions. Typically when CIT reaches 24 h, it is hard to find a patient to accept the offered organ. In most cases, kidneys are discarded after 48 h of CIT. Thus reducing kidney CIT through managerial improvements could be a cost-effective way to improve the current transplantation system and outcomes.

Transplant surgeons and regulators in the US have expressed their concerns regarding high observed kidney discard rates despite the growing waitlist, long wait time, and high waitlist removal rate. Table 2 demonstrates the waitlist and transplant information for the US and Eurotransplant (ET) countries. Eurotransplant is an international nonprofit organization responsible for organ allocation and transplantation in Austria (A), Belgium (B), Croatia (HR), Germany (D), Hungary (H), Luxembourg (LR), Netherlands (NL), and Slovenia (SLO). Even though the number of donated kidneys and transplants performed in 2019 in the US has reached an all-time high, the kidney discard rate of approximately 26% (calculated as the number of deceased kidney transplantations over twice the total number of deceased donors) remains of concern, compared to the ET countries’ discard rate of 20%.

The most common reason for donor kidney refusal and potential discard are concerns about the donor kidney quality. Data shows that transplant surgeons would reject low-quality kidneys for a relatively healthy patient in the hope of receiving a better offer in the future [7]. In addition to the kidney quality, kidney acceptance and discard rates may also be affected by the allocation process itself [8]. Evidence shows that kidneys rejected early in the allocation process are less likely to be accepted later on [9]. Another concern is the increasing risk aversion of transplant centers due to program-specific reports that evaluate post-transplant outcomes. These may provide incentives for the centers to demand higher-quality kidneys. Consequently, they might turn down kidneys adequate for the patient, but that poses a risk of negatively impacting the evaluation of their post-transplant outcomes [8,10,11,12,13,14,15].

Another reason for not observing sufficiently high kidney utilization is the US geographical disparity in kidney transplantation access. Table 3 shows geographic disparities in the number of deceased donors, OPOs, and transplant centers across the 11 regions. Several states such as Wyoming, Idaho, and Montana do not have transplant centers despite their high organ donation rate. Such a variation and difference in OPOs and organ transplant facilities may lead to unfair organ availability, poor access to care, and unnecessary long waiting time for some patients. One of UNOS’s five strategic goals is to provide equity in access to transplant and reduce geographic disparity [16]. To improve the chance of receiving a well-matched donated organ and reduce the long wait time, patients may move to a region with shorter wait times or enlist in multiple transplant centers, typically located in different regions [17]. UNOS has established multiple listing policies that allow patients to be enlisted in more than one transplant center.

Currently, around 4% of the patients waiting for a kidney transplant are multiple-listed, which is the highest rate among all organs [18].

As with any transplant enlisting, the patient must complete evaluation tests and be committed to the transplant center’s regulation, such as the ability to get to the transplant center within a given time.

For enlisting in multiple centers, this process can be quiet costly since most insurance companies may not reimburse the cost of additional evaluations [15,19]. In addition, patients who receive organ transplants are required to take immunosuppression drugs as part of their post-transplant care to make sure their body does not reject a new organ [20]. Therefore, a patient needs to learn if post-transplant care can be transferred to a center closer to her residence. Without policies for adequate financial support for travel expenses, this clearly still poses an issue in terms of equity and fairness that policymakers need to address.

In this paper, we introduce a stochastic simulation model that can be used to analyze the effect of changes to the kidney allocation system and the offering process. The simulation model involves patient’s health, donor-kidney quality represented by Kidney Donor Profile Index (KDPI) [21], donor-kidney quality deterioration due to accumulating CIT during the allocation process and also kidney supply and demand. Furthermore, the model considers the chance that a donor kidney cannot be accepted for other reasons (e.g., short-term sickness of the patient, insufficient surgical resources, cross-matching result). Using model parameters estimated from data provided by UNOS and the Scientific Registry of Transplant Recipients (SRTR), we apply the simulation model to investigate the following two crucial trends to improve donor kidney transplantation rates:

1. Multiple-listing: Transferring to a region with a shorter wait time or waitlisting in multiple regions can help a patient by increasing her chance to receive a kidney transplant earlier. Consequently, the patient can improve post-transplant outcomes due to less health deterioration of staying on dialysis. However, developing a strategy to guide the patient’s decision to transferring or multilisting is not easy. We formulate the decision as a utility maximization problem under a set of budget, distance, and facility constraints at the regional level. Supply and demand vary widely across the 11 US regions and for different blood types. Such a variation results in widely varying wait times, leading to different expected utilities and optimal kidney acceptance strategies (expressed as optimal kidney quality thresholds). To derive a patient’s utility for different regions, we use the simulation model to obtain the utility under individualized optimal kidney transplant acceptance decisions based on the patient’s health status and supply and demand for the patient’s blood type. We use the obtained information to solve the optimization problem and derive an optimal region selection policy.
2. Information technology: Rapid and precise communication between UNOS and transplant centers is necessary to make organ allocation more efficient, which even becomes more critical in the face of multilisted patients. UNOS has the goal to increase the use of information technology in organ allocation and transplantation. They have implemented a secure online-based system that collects data to enhance the transplant system’s capability to improve the patient’s chance of receiving a life-saving organ. As technology has evolved, UNOS also encourages developing and using modern technology such as mobile devices for faster and more efficient consideration of donor’s kidney offers to achieve a higher kidney utilization rate [22].
  For instance, mobile devices will make it easier to collect up-to-date patients’ availability for transplantation (e.g., via an app). Using this information, OPTN will allocate the kidney faster, reducing kidney deterioration and discard. In the ideal case of perfect information, OPTN could find the first patient on the waitlist who will instantly accept the kidney, reducing CIT and discard to a minimum. The presented simulation evaluates the effect of a realistic case of imperfect information sharing.

## 2. Literature Review

In this section, we review both medical and analytical studies about organ transplantation relevant to this paper. For medical papers, we mainly focus on CIT and waiting time on dialysis as two manageable independent risk factors effectively contributing to renal transplant outcomes. For the analytical section, we review papers that fall within one or both of the two research streams concerned with decision-making to accept deceased-donor organs and the allocation process design.

### 2.1. Medical Literature

Several researchers across North America, South America, and Europe have studied the association between CIT and kidney transplant outcomes [23,24]. The analysis done by Nieto-Ríos et al. [25] shows that CIT is an independent risk factor for delayed graft function (DGF). More precisely, the risk of developing DGF increases as CIT surpasses 18 h. However, it does not negatively impact the results in acute rejection or one-year post-transplant graft loss.

A French study by Debout et al. [26] finds that the risk of post-transplant allograft failure and mortality notably increases for each additional hour of CIT. A similar study performed by Valdivia et al. [27] in Andalusia, Spain, confirms that prolonged CIT may impact both patient and graft survival rates. The study suggests that the long CIT may increase the risk of initial poor graft function regardless of both donor and recipient ages. As CIT increase, the chance of DGF also increases. However, the harmful association of prolonged CIT with the risk of DGF is not amplified in older donors (e.g., expanded criteria donors). The study shows that the effect of CIT on acute renal transplant rejection (ARTR) is more noticeable among patients undergoing kidney retransplantation. The analysis also suggests that donated kidneys with CIT of 24 h or longer are at a greater risk of ARTR compared to that of organs with CIT less than 12 h. Koizumi et al. [6] report that regional variations in kidney outcomes have been observed in the US, but the main reason behind these variations is unclear. The study reveals significant cold ischemia time (CIT) variations across regions for donor kidneys. Specifically, they find that regions with longer CIT are more likely to have a lower post-transplant kidney survival rate. They suggest that managerial improvements can be a cost-effective choice to enhance the current transplant system performance and potentiality reduces organ discard rates.

Meier-Kriesche et al. [28] uses the data from the United States Renal Data System Registry (USRDS) to consider the potential association between the wait time and renal transplant outcomes. Their study confirms that long waiting time is a significant risk factor that negatively affects renal transplant’s survival benefit. As a result, they suggest that the earlier the ESRD patients receive a renal transplant, the higher their chances of long-term survival. Meier-Kriesche and Kaplan [29] investigates the importance of wait time on dialysis as the most substantial independent risk factor on kidney transplant outcomes. As part of their analysis, they apply Kaplan–Meier estimates and Cox proportional hazards models on the US renal data system database to explore the effect of wait time on deceased donor kidney results. Their findings show that five- and ten-year graft survival rates are significantly worse among paired kidney recipients who have waited for more than two years on dialysis compared to paired kidney recipients with a wait time of less than half a year.

### 2.2. Analytical Literature

Analytical literature focuses on the design of the kidney allocation process and employs often simulation models. Issues discussed are the effectiveness and equity of the allocation process and the effect the kidney acceptance decision.

To analyze the allocation process used in 2000, Zenios et al. [30] proposes dynamic resource allocation that maximizes the patient’s life expectancy from receiving a kidney transplant while minimizing the inequity between patients. The constructed simulation model shows that the currently-employed organ allocation policy boosts the patient’s quality-adjusted life expectancy and reduces the expected waiting time.

The kidney acceptance decision is central for a whole stream of research. Ahn and Hornberger [31] develops a theoretical model that considers the patient’s health in making an acceptance/rejection decision concerning the quality of offered kidneys. Their analysis reveals that a relatively healthy patient can afford to be selective about the quality of donor kidneys and expect to receive a better post-transplant outcome by accepting a high-quality kidney. The effect of the patient’s choice on the organ allocation system is studied by Su and Zenios [32]. The study introduces a queuing model that analyzes the effects of patient choice on kidney rejection rates by evaluating the waiting system’s performance under both the first-com-first-serve (FCFS) and the last-come-first-serve (LCFS) policies. They conclude that LCFS is more efficient than FCFS. In fact, in contrast to LCFS, the FCFS policy incentivizes patients to refuse low-quality kidneys, resulting in low kidney utilization. On the other hand, the model shows that the LCFS policy obtains optimal organ utilization. In a different study, Su and Zenios [33] investigate the role of patient choice in kidney allocation using a sequential stochastic assignment model. The model addresses the conflict between patient choice and social welfare. The analysis considers two schemes, where the first one assumes that patients have to accept any offered kidney. The first-best solution is to find an allocation policy that maximizes social welfare. By introducing patient choice, the first-best policy is modified to achieve a second-best policy. As a result, an incentive compatibility condition is introduced, which forces the allocation policy to be designed in such a way to assure that patients will accept any kidney offer. Su and Zenios [34] introduce a mechanism design model for organ allocation that takes patient choice into account. Patients state the kidney types (e.g., quality) they desire to receive upon joining the kidney transplant waiting list (not at the time of donor kidney offer) and join the queue that serves the declared kidney type. That way, the model reduces the long searching process by identifying appropriate patients who desire to accept retrieved donor kidneys more effectively.

Fairness and equity are an important topic Bertsimas et al. [35] study geographical disparities in access to deceased donor kidneys. They use a fluid approximation for a queuing model to formulate the optimal way for a patient to be enlisted in the waiting lists of multiple transplant centers. The patient’s objective is to maximize life expectancy while minimizing the congestion cost. By combining analytical, simulation, and numerical results, they show that multiple listing greatly promotes geographical equity and increases the donor kidney supply. Having more donors leads to a higher transplant rate and reduces the patient mortality rate on the waiting list. A few studies [36,37,38] have developed models that enable an incompatible pair of donor-recipient still to receive a living-donor kidney via an exchange with other incompatible donor-recipient pairs. While most existing models aim to maximize the total number of possible kidney exchanges and social welfare, they do not consider the fairness component defined as donor-recipient satisfaction. Lee et al. [39] present a two-stage stochastic programming model that considers fairness in living-donor Kidney Exchange Programs. The study examines multiple scenarios to investigate the effect of fairness on the kidney exchange outcomes. The numerical results show the improvement in the living donor-exchange program’s outcome when fairness is taken into account in matching incompatible pairs. Note that some studies discussed here consider the impact of fairness in living kidney transplantation; however, we focus only on transplants using deceased-donor kidneys.

The simulations presented in the literature use strong assumptions. For instance, Su and Zenios [33] assume that patients have to accept any offered kidney, or in [34] patients cannot change their initially chosen kidney quality. In addition, most simulations typically focus on a single variable. For instance, the study by Ruth et al. [40] focuses on waitlist length. The study proposes a simulation model for the organ allocation process and finds that under the organ allocation conditions in 1985, the waiting list’s length will continue to grow. The simulation model we present in this paper gives a more thorough picture by considering the effect of patient’s decisions, supply and demand in different regions, the efficiency of the allocation process, and the expected effect of post-transplant utility.

## 3. Models Description

In the following sections, we discuss in detail the main components of both simulation and optimization models. The simulation models include patient (organ demand), deceased donor kidney’s arrival (organ supply), the deceased donor kidney consecutive offering process to find the optimal kidney quality threshold, and expected post-transplant utility for a patient with a given health level. We then utilize the simulation model’s output as the coefficients of the optimization model’s objective function to recommend multiple-listing policy and suggests a set of regions that patient can choose.

### 3.1. Simulation Model

**Figure 3:**
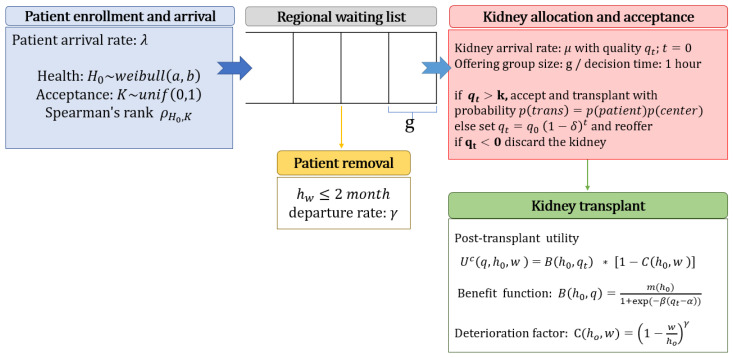
The simulation model for the kidney allocation and acceptance process.

We develop a simulation model that lets the patient identify the optimal kidney quality threshold that maximizes her post-transplant utility. The model parameters depend on the supply and demand of the patients’ region. We simulate the kidney acceptance strategy and the resulting post-transplant utility corresponding to each transplantation. Figure 3 illustrates the simulation process. We discuss the key components in the following sections.

#### 3.1.1. Organ Demand

The demand is represented by patients in the waiting list. We split the patients into several groups of competitive patients who can receive the same type of deceased donor organ depending on blood types and other clinical criteria. We model each group separately. We will consider the interaction between groups (e.g., some patients with blood type AB may receive organs from donors with any blood type) by adjusting organ supply to the individual groups.

**Figure 4:**
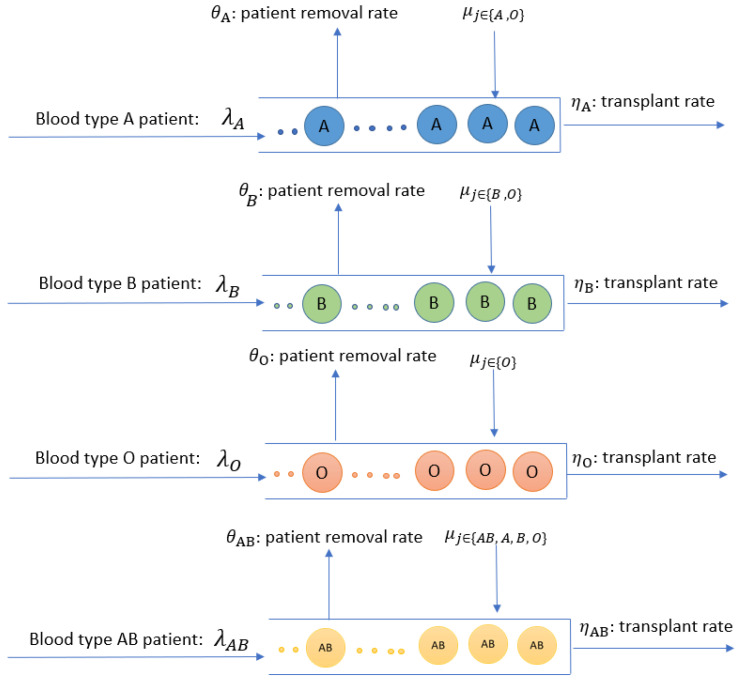
The structure of the queuing model.

Each competitive patient group is modeled by a queue related to blood type *j* where j∈{A,B,AB,O}. Patients can join their matched queue (e.g., based on blood type) with the rate of $\lambda_{j}$ and get served by compatible donors. Compatible kidneys arrive at the blood type *j* queue with a rate of $\mu_{i}$. For instance, for the blood type *A* queue, compatible kidneys are of types *A* and *O*. Patients depart from the waitlist *j* with a transplant rate of $\eta_{i}$ when (1) they accept an offered kidney, or (2) with a rate of $\theta_{i}$ they either get too sick for transplantation or die on the waitlist. The structure of the queuing model is shown in Figure 4.

Following the study in [34], blood type *j* patients arrive according to a Poisson process with the arrival rate of $\lambda_{j}$ to join the waiting list. The patients join the waiting list in the model with an unobservable initial health status $h_{0}$ representing the remaining time they can survive on dialysis when they join. We model the distribution of $h_{0}$ in the patient population using a Weibull distribution. The Weibull distribution is often used in survival analysis to represent time-to-failure since it is able to express failure rates that are decreasing, constant, or increasing over time. The health for a simulated patient, $h_{0}$, is then the realization of a random variable $H_{0}∼\mathrm{Weibull}\left(a,b\right)$, where *a* and *b* are the scale and shape parameters, respectively. Patients depart from the waitlist if either (1) they receive a transplant or (2) they leave the queue due to poor health (or death). Since $h_{0}$ is the time the patient can survive on dialysis when she joins the waitlist (i.e., the index indicates that she waited zero years so far), the actual health after waiting *w* years is $h_{w}=h_{0}-w$ which means the patient will leave the waitlist at the latest when $w=h_{0}$.

#### 3.1.2. Organ Supply

Following [34], compatible deceased donor kidneys arrive at the queue for blood type *j* according to an independent homogeneous Poisson process with arrival rate $\mu_{j}$. OPTN defined a kidney quality metric called Kidney Donor Profile Index (KDPI) that incorporates ten clinical donor factors to rank kidneys according to the estimated post-transplant kidney survival [41]. KDPI considers the following donor characteristics: age, height, weight, ethnicity, whether the donor died due to loss of heart function or loss of brain function, stroke as the cause of death, history of high blood pressure, history of diabetes, exposure to the hepatitis-C virus, serum creatinine (a measure of kidney function). By construction, KDPI is close to uniformly distributed over all kidneys harvested in a given year. Following KDPI, we model the quality of an arriving donor kidney shown with $q_{0}$ as the realization of a random variable Q∼Unif(0,1). We use 0 to represent the lowest and 1 the highest kidney quality, i.e., $q_{0}=1-\mathrm{KDPI}$. When a new donor kidney becomes available in the simulation then the kidney is simultaneously offered to a group of *g* patients with a specified time window to consider the offer and make the acceptance/rejection decision. If nobody in the group of *g* patients can accept the kidney after the allocated time, then the kidney is offered to the next group of *g* patients on the waitlist. The allocation process continues until the organ is either accepted by a patient or discarded (due to an unsuccessful search or organ placement). For the current donor kidney shortage, we have $\mu_{j}<\lambda_{j}$, i.e., kidneys arrive at a lower rate than new patients. Patient removal due to health or death keeps the queue at a finite size. Longer waitlists result in longer wait times and more health deterioration for the patients. In turn, this increases the removal rate (patients leaving without receiving a transplant). The queue length stabilizes at the equilibrium where the transplant rate plus the patient removal rate match the patient arrival rate.

#### 3.1.3. Kidney Acceptance/Rejection Decision

Over time, as the process of donor kidney offering continues, the kidney accumulates CIT and its quality deteriorates. We model this deterioration as $q_{t}=f\left(q_{0},\delta ,t\right)$. In this equation *t* represents the accumulated CIT and $q_{0}$ represents the kidney quality at the time of recovery when t=0. Variable δ represents a kidney quality deterioration factor. We require that the quality function *f* is decreasing in δ and *t*, i.e., $\frac{\partial f(q_{0},\delta ,t)}{\partial \delta }<0$ and $\frac{\partial f(q_{0},\delta ,t)}{\partial t}<0$. In the simulation model, we measure time as multiples of the time allowed for one round of offers. If the patients have one hour to decide, then *t* represents accumulated CIT in hours. We model the kidney accept/reject decision by the patient and the subsequent transplantation in two steps. First, the patient uses a threshold strategy to decide if an offered kidney is acceptable. The patient would accept the offer if $q_{t}\geq k$, where *k* is the kidney quality threshold decided on by the patient and the surgeon. For acceptable kidneys, we consider several factors related to the patient’s health and the transplant center. In the simulation model, we use the probability of the transplant being performed given an acceptable kidney is offered as

$$p\left(\mathrm{transplant}\;|\;q_{t}\geq k\right)=p\left(\mathrm{patient}\;\mathrm{factors}\right)\;p\left(\mathrm{center}\;\mathrm{factors}\right),$$

where p(patientfactors) represents the patient’s specific medical situation and any fact that the patient or the surgeon may decide against the kidney for reasons not explained purely by kidney quality (e.g., the patient is temporary set inactive on the waitlist, unfavorable cross-matching result). The probability p(centerfactors) represents the transplant center’s readiness (e.g., availability of staff, operating rooms, etc.) as well as considerations of the impact of the transplantation on the center’s performance evaluation. The patient chooses her decision threshold *k* in consultation with the surgeon. Such a threshold will be influenced by the patient’s health $h_{0}$ since a patient who has more time left on dialysis will wait for a better quality kidney. We model this relationship in the simulation by choosing *k* for each patient from a random variable K∼Unif(0,1) which is correlated with the patient’s $h_{0}$ represented by a Spearman’s rank correlation coefficient of $\rho_{H_{0},K}$.

#### 3.1.4. Patient’s Post-Transplant Utility

If the patient accepts the deceased donor kidney offer and transplantation occurs, a patient receives post-transplant utility. The post-transplant utility depends on the kidney’s quality at transplantation time $q_{t}$, and the patient’s wait time *w* resulting in a health status of $h_{w}=h_{0}-w$. The post-transplant utility can be broken down into two components

(1) $$U\left(q_{t},h_{0},w\right)=B\left(h_{0},q_{t}\right)\;D\left(h_{0},w\right),$$

where B(·) represents the benefit for the patient depending on the kidney quality, and D(·) accounts for the deterioration of the patient on the waitlist. Breaking the utility into these two components has benefits for estimating parameters from data. The function B(·) can be seen as the patients benefit if she would receive a kidney with quality $q_{t}$ without waiting. The benefit function needs to satisfy that it increases with patient health $h_{0}$ and the kidney quality, i.e., $\frac{\partial B(h_{0},q_{t})}{\partial h_{0}}>0$ and $\frac{\partial B(h_{0},q_{t})}{\partial q_{t}}>0$. D(·) represents a cost in the form of a deterioration factor due to waiting *w* for the kidney. The cost function needs to increase as wait time increases and decreases with the patient health., i.e.,$\frac{\partial D(h_{0},w)}{\partial h_{0}}>0$ and $\frac{\partial D(h_{0},w)}{\partial w}<0$.

A common way to define functions like B(·) is in the form of a logistic regression for survival proposed by Cox [42] which models the conditional odds of dying at any time point given survival up to that point as

(2) $$B\left(h_{0},q_{t}\right)=\frac{m(h_{0})}{1+exp(-\beta \left(q_{t}-\alpha \right))},$$

where $m(h_{0})$ indicates the transplant outcome for a patient with health level $h_{0}$ who received a perfect kidney ($q_{t}=1$) right away (w=0). Naturally, $m(h_{0})$ is increasing with the patient’s health $h_{0}$. We use for D(·) the functional from

(3) $$D\left(h_{0},w,\gamma \right)={\left(1-\frac{w}{h_{0}}\right)}^{\gamma },$$

where γ controls the rate of deterioration. The deterioration factor equals one (i.e., no deterioration) when the wait time is zero (w=0). If the patient waits for a very high-quality kidney and runs out of time (i.e., $w=h_{0}$), then the deterioration factor becomes zero. The chosen functional form is vert flexible and can express linear deterioration (γ=1), slowing down deterioration (γ>1), and increasing deterioration (γ<1). By estimating parameters from data and using simulation optimization, we can find for each patient the the optimal kidney quality threshold $k^{*}$ that maximises the post-transplant utility.

### 3.2. Region Selection and Multiple Listing Optimization Model

A patient can improve her chances of receiving a transplant by moving to a different region (region selection) or by listing in transplant centers in multiple regions. To help the patient to identify a set of regions for multiple-enlisting, we utilize the simulation model to calculate the optimal threshold policy parameter $k_{i}^{*}$ and the maximum expected utility that a patient is likely to obtain from a transplant in each region. This is represented by 11 utility values $U_{i}\left(k_{i}^{*},h_{0},w\right)$, i∈1,2,…,11. For simplicity, we write $U_{i}\left(k_{i}^{*}\right)$ to represent the post-transplant expected utility for a patient with given $h_{0}$ and *w*. Region selection is now done by picking the region with the largest utility.

For multiple listing, we represent the action of enlisting in region *i* by the binary decision variable

(4) $$r_{i}=\left\{\begin{array}{cc} 1 & \mathrm{if}\;\mathrm{region}\;i,\;\mathrm{and}\\ 0 & \mathrm{otherwise}. \end{array}\right.$$

The patient has 11 decision variables, one for each region. We assume that the patient will want to increase her chances by listing in the best regions with the highest expected utility given a set of constraints. This can be formulated as the following optimization problem.

(5) $$\begin{array}{cc} \; & \max\;\sum_{i\in I}U_{i}\left(k_{i}^{*}\right)r_{i}\\ \; & s.t.\\ \; & \sum_{i\in I}c_{i}r_{i}\leq C\\ \; & d_{i}r_{i}\leq D\\ \; & p_{i}r_{i}\geq P\\ \; & r_{i}=0\;\mathrm{or}\;1 \end{array}$$

Summing the region utility makes sure that the regions with the largest utilities are included in the solution. The first constraint makes sure that the solution satisfies the total budget *C* of the given patient. The second constraint considers the maximum distance *D* the patient can travel to get to the transplant center in time. The third constraint considers the patient’s expectation about the region’s performance *P*, and finally, the last constraint restricts $r_{i}$ to be 0 or 1. Since the number of regions is small, with only 11, this problem can be solved by enumeration.

## 4. Applications and Numerical Results

We start this section with estimating the parameters needed for the simulation model and then present how the model can be used for two applications. The first application illustrates how the model can provide a strategic guideline to support a patient’s choice for moving to a different region or enlisting in multiple regions.

The second application analyzes the potential benefits of using modern information shared technology (e.g., via smartphone apps) to improve social welfare through increasing patient’s post-transplant utility and kidney utilization rates.

### 4.1. Parameters Estimation

We use data from UNOS and SRTR to estimate model parameters. We extract UNOS data for the year 2019 to estimate waitlist additions and donor kidney supply. For wait time calculation, we use values reported by SRTR. The SRTR data system contains detailed medical and demographic data for all donors, waitlisted patients, and transplant recipients in the US. The used dataset consists of more than 400,000 patients who are first-time recipients of deceased donor kidney transplants between October 1987 and the end of 2019.

The annual reports by SRTR and UNOS provide information on organ arrivals and waiting list activity (e.g., patient’s addition and removal statistics). We use this data to estimate $\lambda_{j}$ and $\mu_{j}$ in each period.

An important factor for estimating the kidney arrival rate to a blood type *j* queue is blood type compatibility between patients and donors. According to the blood type compatibility criteria, donors with blood type O are universal donors whose kidney organs can be offered to patients of all blood types. On the other hand, donors with blood type AB can donate their kidneys to only blood type AB patients while they are universal recipients from all blood types. Table 4 shows the blood type compatibility for a kidney transplant in detail. This paper only reports results for blood type A. The results for other blood types can be obtained similarly. Table 5 shows donors and patient arrivals for blood type A.

Based on SRTR data blood type A patients receive on average 94% and 6% of the organs from donors of blood types A and O, respectively, which is reflected in the kidney supply parameter $\mu_{j}$ in Table 5. Following the current offering scheme used by OPTN in the US, we use the patient group size of g=5 in our simulation model. We set the kidney degradation rate δ to 5% according to the reports that organs are rarely used after a CIT of 48 h [6]. At δ=0.05, the quality of the kidney deteriorates to ${\left(1-0.05\right)}^{48}=8.5\%$ of its initial quality after 48 h. Based on discussions with a medical collaborator, we use a transplantation probability p(transplant)=0.8 for all regions. A probability for each region could also be estimated from data, but information on rejections of kidney offers is currently not available to us. The parameters α, β, and γ for the benefit function $B(h_{0},q_{t})$ and the cost factor $C(h_{0},w)$ can be estimated if the outcome data including the post-transplant survival is available. However, since our dataset does not include these data, we use α=0.4, β=8 and γ=0.5 in our simulation. We add patients to the waitlist with a health $h_{0}$ drawn from a random variable $H_{0}$ with a Weibull distribution. We use a scale parameter a=8 and a shape parameter b=2 to get an average health of close to 7 years and around 90% of the population below 12 years. We use a Spearman’s rank correlation $\rho_{\left(H_{0},K\right)}$ of 0.2, close to the correlation between the accepted kidney quality and the patient health observed in the data.

### 4.2. Region Selection and Multiple Listing

To illustrate the region selection approach, we report the results for a target patient with blood type A, one life-year on dialysis ($h_{0}=1$) who is currently in position 100 of the waitlist. We fill the waitlist with randomly generated patients (whose health is drawn from a Weibull distribution with a correlated policy threshold). We perform the same simulation 100 times each for the decision threshold values k∈{0,0.1,0.2,⋯,0.9} and average the results of the 100 runs to estimate the expected utility for each threshold.

Table 6 reports the results for the optimal threshold, $k_{i}^{*}$, resulting in the largest expected post-transplant utility, $U_{i}\left(k_{i}^{*}\right)$, for each region. The kidney arrival rate to the queue (blood type A waitlisted patients) per year is $\mu_{A}$ and $q_{t}$ is the average transplanted kidney quality. For instance, if the target patient is enrolled in region 6, a threshold of k=0.65 is optimal, which leads to a utility of 9.6 years. In contrast, if she is enlisted in region 2, the optimal decision can be as high as 0.85 with a utility of 13.22 years.

Table 7 represents the estimated data we used in our optimization model to find a set of feasible regions for a blood type A patient assumed to be currently living and enlisted in region 6. We use a UNOS dataset to estimate the expected wait time and the 5-year survival rate for such patient across all 11 regions. In each region, we choose a major city and estimate its corresponding monthly cost of living using the city’s cost of living index. The evaluation cost is defined as the product of the total expected number of evaluations until kidney transplantation and cost per evaluation. The expected number of evaluations is estimated based on a 6-month reevaluation policy mandated by most transplant centers. In general, the patient is responsible for paying for the periodic evaluation cost if she wishes to be enlisted in more than one region since most insurance policies cover the periodic evaluation cost of only one registration. The total cost is calculated as follows: the total amount of money a patient has to pay (number of evaluations times cost of evaluation) plus the cost of traveling to and staying in another region for three days.

As an example, here we assume the patient has a budget of *C* = $15,000. She also can travel as far as D=1500 miles, and her minimum expectation from a region’s performance is 75% of five-year survival. Intending to maximize her post-transplant outcome under these three constraints, our model finds that besides home-region 6, the patient can also be enlisted in regions 5, 4, or 8, with region 5 providing the highest expected utility. OPTN is committed to provide equity in access to transplants and reduce geographic disparities [16]. Under free multiple listing, patients have an incentive to enroll in as many regions as they can, given their budget constraints. This means that access to transplantation is affected by the patient’s financial resources, which may pose a problem in terms of equity and fairness. However, in the long run, free region selection and multiple listing can reduce geographic disparities. More patients will enlist in regions that are currently offering higher utility, leveling out demand disparities, and reducing the utility gap. At that point, the advantage of having more budget to enlist in multiple regions will diminish, leading to a more equitable situation. In the short term, equity needs to be ensured temporarily by appropriate policies to make multiple listings available to more patients.

### 4.3. The Effect of Information Sharing on Allocation Efficiency

One of the initiatives in the OPTN Strategic Plan (2018–2021) [16] under the goal of increasing the number of transplants is to pursue system tools for more efficient donor/recipient matching. Such tools include tools for information sharing, which means the transplantation center and the patient share up-to-date information with OPTN, has the potential to speed up the kidney allocation process and thus reduce cold ischemia time (CIT) and kidney discard rate.

Information that can be shared includes:

1. The patient’s acceptance threshold *k*: Each patient reports her kidney quality acceptance threshold *k* decided by herself and her physician.
2. Any additional decision criteria used by the patient: The patient’s and surgeon’s decision can be affected by information not included in the kidney quality assessment (KDPI). Having more standardized quality parameters, where the patient can prespecify what she accepts, would improve kidney allocation. Under complete information, OPTN could instantly identify the patients who would accept the kidney and save valuable CIT.
3. The patient’s current availability: An up-to-date indication if the patient can currently receive transplantation. Factors include current health and traveling.
4. The transplant center’s availability: Considers the availability of the transplant center’s facility such as prepared operating rooms, surgeons, nurses, and staffs for performing the surgery on time.

Under perfect information, OPTN would have access to up-to-date information about all patients’ acceptance thresholds *k*, any additional requirement for the organ, and if the patient and center are available. Therefore, OPTN could directly identify the first patient on the waitlist who will accept and receive the transplant. This will effectively reduce CIT, i.e., *t*, to the minimum needed to extract the organ and perform the transplantation. In the simulation model, perfect information can be expressed by setting *g* to the waitlist’s length, indicating that the whole waitlist is searched for a matching patient instantaneously. Assuming perfect information is unrealistic for many reasons. For example, technical issues may impact information availability and patients or centers may not keep all the information constantly up-to-date. A more realistic setting is improved but still imperfect information sharing. More information means that patients can be identified faster using the shared information. We express this fact in the simulation model by an increased number of patients that can be searched per hour (i.e., an increase in *g*).

To illustrate the effect of improved information sharing, we report the results for patients of blood type A in region 6. We initialize the waiting list with 1000 patients and run the simulation until the waitlist length stabilizes around 1800 patients (200 months). We report results after this warm-up period averaged over 300 months. The baseline is the currently used group size of g=5. We vary *g* to represent varying levels of information sharing.

Table 8 shows the impact of information sharing expressed by how many patients on the waitlist can effectively be considered per hour. At the baseline group size of five, the accepted kidney’s average quality is 0.66, leading to the average utility of 10.76 years per transplanted patient. The kidney can travel as far as 45 patients on the waitlist and is accepted on average by the sixth patient. Table 9 presents kidney utilization and waitlist mortality rates, in addition to the transplant rate. The kidney utilization rate increases significantly as *g* increases.

**Figure 5:**
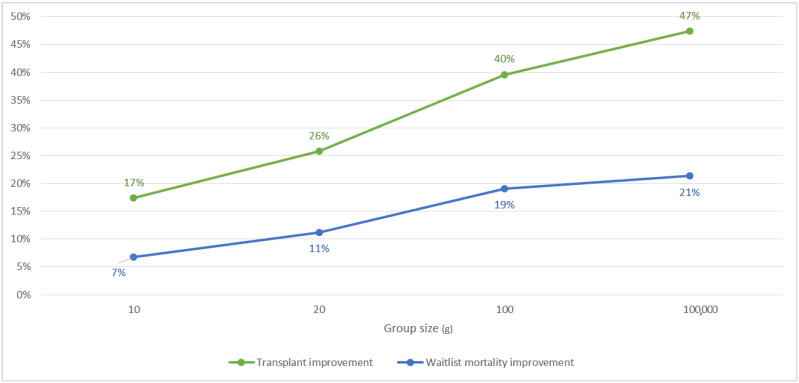
Kidney transplant and waitlist mortality rates improvements due to information sharing compared to the baseline of g=5.

As Figure 5 demonstrates, the improvement in the kidney transplant rate is 17% for doubling the speed of the offering process g=10, and it reaches 47% when perfect information is available. On the other hand, the waitlist mortality rate decreases by 7% when g=10, and the reduction can be as high as 21%. The simulation illustrates the effect on efficiency that information sharing can bring to the allocation process.

Information sharing can be implemented in many ways, using current technology. Examples include using apps and medical wearable devices to track the patient’s availability close to real-time. Standardized interfaces between the transplant centers’ information system and OPTN can be used to manage center availability. Rolling out these technologies will take time, but the results presented in this simulation study indicate that the potential payoff is significant with the potential to reduce kidney discard rates to a minimum.

## 5. Concluding Remarks

This research’s first contribution is developing a simulation model that provides an optimal deceased donor kidney acceptance guidance for decision-makers (patients and surgeons). The major challenge of modeling organ acceptance/rejection is incorporating real-world conditions and situations to make a crucial life-saving decision. For this reason, our primary intention as the main novelty of this work is to recognize, aggregate, and implement different essential elements that contribute to kidney selection criteria. The proposed model allows for diversity in patients’ health and kidney’s quality, as well as their correlation. Moreover, we include the quality deterioration of kidneys caused by accumulating CIT as the allocation process goes on. In addition to all aforementioned elements, we also incorporate patient health and availability together with human and facility resources to propose an optimal transplant solution.

The proposed model can be used to investigate how different policy choices can affect the strategic goals stated by OTPN [16]. We illustrated this with two applications. First, we showed how the model can be applied to inform patients’ decisions regarding multiple-enlisting given cost, distance, and care quality constraints. While multiple listing in the short-term can pose challenges to equity between patients based on financial resources, it has the potential to even out geographical disparities in access to transplants and thus increase equity.

A second illustration draws attention to the social welfare aspect of kidney transplantation rather than focusing on finding an optimal solution as considered in the first application. We compare the social welfare results (i.e., donor kidney utilization and system-wide post-transplant utility) for several levels of available information, ranging from no information to perfect information. Increased information leads to faster kidney assignment and a reduced kidney discard rate. Increasing the transplant rate improves social welfare utility and reduces the length of the kidney transplant waiting list, time, and mortality rate. Policymakers can use these results to motivate the value of modern information technology to collect the needed information and show the importance of designing incentive structures that encourage timely information sharing by patients and centers. For example, designing an organ transplantation application for a smartphone device can provide a safe, easy, and fast way to submit and update the required information in a timely fashion. The policymaker may wish to establish a ground rule that all patients and transplant centers need to follow to receive offers. For instance, using mandatory app technology and service, transplant centers can revise or verify their submitted data regularly (e.g., every day) after a patient’s position on the waitlist passes a certain threshold. The proposed model is simple and flexible enough to be easily adapted to investigate many other aspects of the kidney assignment process.

## Figures and Tables

**Table 1:** One and five-year transplant outcomes across 11 regions in US.

One-Year Post Transplant Patient and Graft Survival Rate				
Region	Patient Survival Rate	95% Confidence	Graft Survival Rate	95% Confidence
1	96	(94.9, 96.8)	93	(91.6, 94.1)
2	95.7	(95.1, 96.2)	92	(91.3, 92.7)
3	96.4	(95.9, 96.8)	93.5	(92.8, 94.0)
4	95.8	(95.2, 96.4)	93.1	(92.3, 93.8)
5	97	(96.6, 97.4)	94	(93.5, 94.5)
6	98	(97.2, 98.5)	96.3	(95.3, 97.1)
7	95.3	(94.5, 96.0)	93	(92.0, 93.8)
8	97	(96.3, 97.5)	93.7	(92.8, 94.5)
9	95.2	(94.5, 96.1)	91.6	(90.5, 92.5)
10	95.7	(95.0, 96.3)	92.6	(91.7, 93.4)
11	96.6	(96.0, 97.0)	93.3	(92.6, 94.0)
1	82.1	(80.0, 84.0)	75	(72.7, 77.1)
2	80.7	(79.6, 81.8)	69.9	(68.6, 71.1)
3	84.7	(83.8, 85.6)	76.2	(75.1, 77.3)
4	84.6	(83.4, 85.8)	75.1	(73.6, 76.5)
5	85.5	(84.6, 86.4)	79	(78.0, 80.0)
6	88.8	(87.0, 90.3)	82.7	(80.7, 84.6)
7	82.3	(81.0, 83.6)	73.8	(72.3, 75.2)
8	84	(82.6, 85.4)	75.5	(73.8, 77.0)
9	80	(78.5, 81.5)	69.8	(68.1, 71.5)
10	81.7	(80.3, 83.0)	71.9	(70.3, 73.4)
11	82.5	(81.4, 83.6)	72.3	(71.0, 73.5)

**Table 2:** 2019 Snapshot of US and Eurotransplant (ET) countries with donation rates, waitlist, and transplantation activities. See the footnote on page 4 for acronym definitions of ET countries. Note that most deceased donors can donate both kidneys and therefore the number of deceased kidney transplantation is more than the total deceased donors.

	US	A	B	D	H	HR	NL	SLO	Total ET
Total deceased donors	11,152	197	276	904	167	127	289	50	2010
Deceased kidney transplantation	16,534	298	426	1536	281	158	445	53	3197
Waitlist mortality	4012	30	29	399	52	10	67	1	588
No longer eligible for transplant	4285	23	43	187	24	7	112	9	405
Waiting list addition	34,480	487	616	2797	376	223	1510	77	6086
Current kidney waiting list length	99,122	616	870	6881	824	231	803	95	10,320

**Table 3:** Kidney transplant waitlist information and the number of OPOs and transplant centers across the 11 regions.

Region	1	2	3	4	5	6	7	8	9	10	11	All Regions
Deceased donor	399	1373	1639	1196	1758	475	833	854	461	953	1211	11,152
Transplant (from deceased donor)	597	1885	2491	1632	2771	605	1122	1151	1039	1350	1891	16,534
Current waitlist length	4846	12,580	12,818	10,447	22,366	2512	7258	3741	7696	5348	9523	99,122
Waitlist addition	1546	4178	4687	3990	5884	1007	2741	1771	2369	2445	3862	34,480
Waitlist removal (death)	197	523	560	387	1021	49	268	141	327	201	338	4012
Waitlist removal (health)	184	551	588	530	677	159	375	173	264	282	502	4285
Numbers of OPOs	2	5	10	4	8	3	4	5	4	6	7	58
Numbers of Transplant centers	14	36	30	29	31	10	23	18	16	20	24	251

**Table 4:** Blood type compatibility for kidney transplantation.

Blood Type	% of US	Can Donate Kidney to	Can Receive Kidney from
O	45%	O, A, B, AB	O
A	40%	A, AB	O, A
B	11%	B, AB	O, B
AB	4%	AB	O, A, B, AB

**Table 5:** Estimation of annual kidney supply $\mu_{j}$ and patient arrival rates $\lambda_{j}$ for blood type A waitlisted patients over the 11 US regions (2019).

Region	Kindey Arrival Rate to Blood Type A Queue ($\mu_{A}$)	Blood Type A Patient Arrival Rate ($\lambda_{A}$)
1	229	536
2	748	1774
3	892	1680
4	587	1154
5	882	1914
6	270	378
7	461	1008
8	436	690
9	220	735
10	548	1040
11	590	1089

**Table 6:** Optimal post-transplant utility $U_{i}\left(k_{i}^{*}\right)$ under the optimal decision threshold in different regions for a blood type A patient in waitlist position 100.

Region	Kidney Arrival Rate	Optimal Kidney Quality	Average Life Years Gain
b	to Blood Type A Queue ($\mu_{A}$)	Acceptance Threshold ($k_{i}^{*}$)	form Transplant ($U_{i}\left(k_{i}^{*}\right)$)
1	229	0.60	9.12
2	748	0.85	13.23
3	892	0.85	13.57
4	587	0.80	12.81
5	882	0.85	13.50
6	270	0.65	9.65
7	461	0.75	12.00
8	436	0.75	11.90
9	220	0.60	8.9
10	548	0.80	12.70
11	590	0.80	12.84

**Table 7:** Illustration of the region selection process for multiple-region listing.

Region	Cost of Living per Month	Expected Wait Time in Years	Evaluations	Evaluation Cost	Survival Rate (5 Year)	Distance (Air Miles)	$U_{i}\left(k_{i}^{*}\right)$
1	$2454	2.4	4	$6381	73%	2200	9.1
2	$1879	1.8	3	$4614	70%	1904	13.2
3	$1637	1.9	3	$4541	76%	1706	13.6
4	$2328	1.4	2	$3166	75%	1160	12.8
5	$2130	2.5	5	$7815	79%	374	13.5
6	$2413	1.6	3	$0	83%	0	9.7
7	$2686	2.3	4	$6474	74%	670	12.0
8	$2869	1.4	2	$3274	76%	569	11.9
9	$2705	2.2	4	$6482	69%	1195	8.9
10	$2005	1.8	3	$4651	72%	565	12.7
11	$2706	1.4	2	$3241	72%	941	12.8

**Table 8:** The effect of information sharing on patient’s post transplant utility based on region-6 kidney supply and demand.

Group Size *g*	Waitlist Position (Mean)	Waitlist Position (Max)	Average *q*	Average Patient Utility (Year)
5	6.00	45	0.66	10.76
10	8.00	80	0.62	11.20
20	10.00	119	0.60	11.14
100	22.00	344	0.57	11.35
100,000	58.00	1383	0.55	11.50

**Table 9:** Kidney utilization, discard, waitlist removal, and transplant rates in region 6.

Group Size *g*	Kidney Utilization Rate	Kidney Discard Rate	Waitlist Removal Rate	Kidney Transplant Rate
5	85.0%	15.0%	8.9%	17%
10	90.9%	9.1%	8.3%	20%
20	94.3%	5.7%	7.9%	21.5%
100	98.5%	1.5%	7.3%	23.8%
100,000	99.98%	0.02%	7.1%	25.2%

## Data Availability

The data set used in this analysis can be obtained through UNOS (https://unos.org/) and SRTR (https://www.srtr.org/).
